# Levosimendan versus dobutamine in septic cardiomyopathy: a randomized clinical trial on cardiac function and safety

**DOI:** 10.3389/fcvm.2025.1641604

**Published:** 2025-09-19

**Authors:** Feng Zhao, Haolei Wei, Leqing Lin, Hui Wang, Zhuxian Zhang, Liang Guo

**Affiliations:** ^1^ Department of Emergency Medicine, Tongde Hospital of Zhejiang Province, Hangzhou, Zhejiang, China; ^2^ Department of Intensive Care Unit, Affiliated Hospital of Hangzhou Normal University, Hangzhou, Zhejiang, China

**Keywords:** sepsis, septic cardiomyopathy, levosimendan, dobutamine, cardiac function

## Abstract

**Objective:**

This study aims to evaluate the clinical efficacy, safety, and impact on outcomes of levosimendan compared with dobutamine in patients with septic cardiomyopathy.

**Methods:**

A randomized clinical trial was conducted in patients with septic cardiomyopathy between December 2022 and March 2024. Eligible patients received either levosimendan or dobutamine in addition to standard sepsis treatments. Baseline characteristics, laboratory parameters, pulse index continuous cardiac output, clinical outcomes, and adverse reactions were recorded and compared between the two groups.

**Results:**

A total of 50 patients were analyzed, with 25 patients in each group. The mean age was 76.4 (±12.3) years, and 28 patients (56%) were male. Baseline characteristics were comparable between groups. Following treatment, improvements were observed in both groups in left ventricular ejection fracture and levels of cardiac troponin I, B-type natriuretic peptide, cardiac index (CI), lactate, and norepinephrine infusion rate(all *P* < 0.05), with significantly greater improvements in the levosimendan group (*P* < 0.05). Additionally, the CI was higher in the levosimendan group compared to the dobutamine group (*P* < 0.05). No statistically significant differences were observed between groups in other pulse index continuous cardiac output variables, laboratory tests, clinical outcomes, or adverse reactions.

**Conclusions:**

In patients with septic cardiomyopathy, levosimendan treatment resulted in greater improvements in cardiac function, hemodynamic stability, and tissue perfusion compared with dobutamine, without an increase in adverse reactions. Further studies are needed to evaluate the long-term effects of levosimendan on clinical outcomes in this patient population.

**Registration number:**

ChiCTR2500101261.

## Introduction

Sepsis is a life-threatening multi-organ dysfunction resulting from a dysregulated host response to infection (1). Septic cardiomyopathy refers to acute cardiac dysfunction unrelated to myocardial ischemia that occurs in sepsis patients. Its prevalence in septic patients was estimated at up to 70% (2), with a mortality rate as high as 70%–90% (3). Currently, the treatment for septic cardiomyopathy is to improve the cardiac function. In clinical practice, the most commonly applied positive inotropic drug in septic shock patients is dobutamine (4). Dobutamine can increase myocardial contractility by directly activating cardiac β1 receptors and augment cardiac output, reducing peripheral vascular resistance and ventricular filling pressures, and promoting atrioventricular node conduction. However, dobutamine can increase heart rate and myocardial oxygen consumption, with potential negative impacts on patient outcomes (5). There is no consensus on the selection of inotropic drugs for septic cardiomyopathy.

As a novel cardiotonic drug, levosimendan binds to cardiac troponin C in a calcium-dependent manner. Levosimendan can enhance the calcium sensitivity of contractile proteins and produce positive inotropic effects. In addition, levosimendan can induce diastole in coronary resistance vessels and dilate systemic venous volume vessels by opening adenosine triphosphate-sensitive potassium channels in vascular smooth muscle (6). Theoretically, levosimendan has the advantages of maintaining systemic and pulmonary circulation without elevating intracellular calcium ion concentration, accelerating ventricular rate, and increasing myocardial oxygen consumption. However, the potential benefits or limitations of levosimendan have not been confirmed in the clinic. Gordan et al. reported that the addition of levosimendan in patients with septic shock failed to improve the morbidity and mortality but led to supraventricular tachycardia and difficulty in weaning from mechanical ventilation (7). Subsequent subgroup analyses also suggested that the addition of levosimendan to standard sepsis treatment was not associated with improved organ dysfunction and reduced mortality in patients with cardiac insufficiency (8). However, a meta-analysis, including 192 patients with sepsis-associated cardiac insufficiency, found that levosimendan improved cardiac function and reduced extravascular lung fluid and lactic acid better than dobutamine (9).

In this study, we compared the clinical efficacy, safety, and outcomes of levosimendan vs. dobutamine treatments in patients with septic cardiomyopathy to provide evidence-based clinical application of levosimendan in this patient population.

## Materials and methods

### Study design and participant selection

A randomized clinical trial was conducted in patients with septic cardiomyopathy admitted to the Department of Intensive Care Medicine at the Affiliated Hospital of Hangzhou Normal University, China, between December 2022 and March 2024. The study protocol was approved by the university's ethics committee and registered in the Chinese Clinical Trial Registry (registration number: ChiCTR2500101261). Written informed consent was obtained from all study participants.

The inclusion criteria were (10) as follows: (1) diagnosis of sepsis or septic shock based on the Sepsis-3.0 criteria (2) left ventricular ejection fraction (LVEF) <45%, or > a decrease of  10% from baseline; (3) cardiac troponin I (cTnI) >0.06 ng/ml and B-type natriuretic peptide (BNP) >100 pg/ml; and 4) age ≥18 years.

Exclusion criteria included: (1) confirmed diagnosis of acute coronary syndrome within two weeks before or after sepsis diagnosis; (2) history of chronic heart failure; (3) history of cardiac surgery or pacemaker implantation; (4) malignant arrhythmias; (5) hypertrophic or restrictive cardiomyopathy, or severe valvular stenosis or regurgitation; (6) severe hepatic or renal dysfunction; and (7) use of positive inotropic agents within one week prior to the trial. Additionally, patients who underwent cardiopulmonary resuscitation, electrical defibrillation, extracorporeal membrane oxygenation, or intra-aortic balloon pump after trial initiation were excluded from the analysis.

### Study protocol

The participants were randomly assigned to either the levosimendan group or dobutamine group. All of them were treated following the standard sepsis guidelines, including antibiotics, fluid resuscitation, and supportive care, and were monitored by the pulse index continuous cardiac output (PiCCO). In the levosimendan group, levosimendan was initiated at 0.1 μg/kg/min and titrated to a range of 0.05–0.2 μg/kg/min to maintain the mean arterial pressure above 65 mmHg. The treatment was continued for 24 h. In the dobutamine group, dobutamine was initiated at 5 μg/kg/min and titrated within a range of 2.5–20 μg/kg/min to maintain mean arterial pressure (MAP) above 65 mmHg. Treatment was continued for 72 h.

If adverse reactions related to the study drugs occurred, symptomatic management was implemented. This included maintaining adequate volume status, adjusting doses of vasoactive and sedative–analgesic agents, replenishing electrolytes, correcting acid–base imbalances, and optimizing respiratory parameters. If symptoms persisted after 2 h of such interventions, the dose of levosimendan or dobutamine was appropriately reduced. The observation was continued for an additional two hours. If symptoms resolved, the drug dose was re-adjusted to the original level. If symptoms remained unresolved, the study drug was discontinued, the trial was terminated for that patient, and further symptomatic treatment was provided to ensure patient safety.

If there was suspected excessive fluid infusion prior to the admission to the intensive care unit (ICU), restricted fluid intake would be applied. Patients with two or more of the following characteristics would receive intravenous diuretics, (1) clinical symptoms or signs of increased heart rate, shortness of breath, edema, or pink frothy sputum, (2) chest imaging showing hilar butterfly shadow, pleural effusion, (3) negative passive leg-lifting test, (4) significant increase in BNP, (5) ultrasound demonstrating right heart or inferior vena cava dilation.

If a patient's daily urine output remained <800 ml after a daily dose of 80 mg furosemide, with significantly elevated serum creatinine level, continuous renal replacement therapy would be initiated and the patient would be excluded from this trial.

### Data collection

Baseline information, including sex, age, height, weight, the primary site of infection, sequential organ failure assessment (SOFA) score, and acute physiology and chronic health evaluation (APACHE) II score were recorded. Clinical and laboratory parameters, including heart rate (HR), MAP, norepinephrine dosage (NED), lactate, left ventricular ejection fracture (LVEF), cTnI, BNP, cardiac index (CI), central venous pressure (CVP), global end-diastolic volume index (GEDVI), extravascular lung water index (EVLWI), systemic vascular resistance index (SVRI), white blood cell (WBC) count, C-reactive protein (CRP), and procalcitonin (PCT) were measured at baseline and 24, 48, and 72 h after treatment initiation. Additionally, the length of ICU stay, duration of mechanical ventilation, 28-day mortality, SOFA score at 72 h post-treatment and APACHE II score at 72 h post-treatment were recorded. Adverse reactions during treatment were also documented.

### Statistical analysis

All statistical analyses were performed in SPSS (version 27.0, IBM, New York, USA). The continuous data were presented as mean ± standard deviation or median with interquartile range and were compared by either t-test, ANOVA, Mann–Whitney, or Kruskal–Wallis test, depending on the normality test results. The categorical data were presented as numbers with percentages and compared using the Chi-square test. A *P* < 0.05 was considered statistically significantly different.

## Results

### Patient enrollment and baseline characteristic comparisons

**Figure 1:**
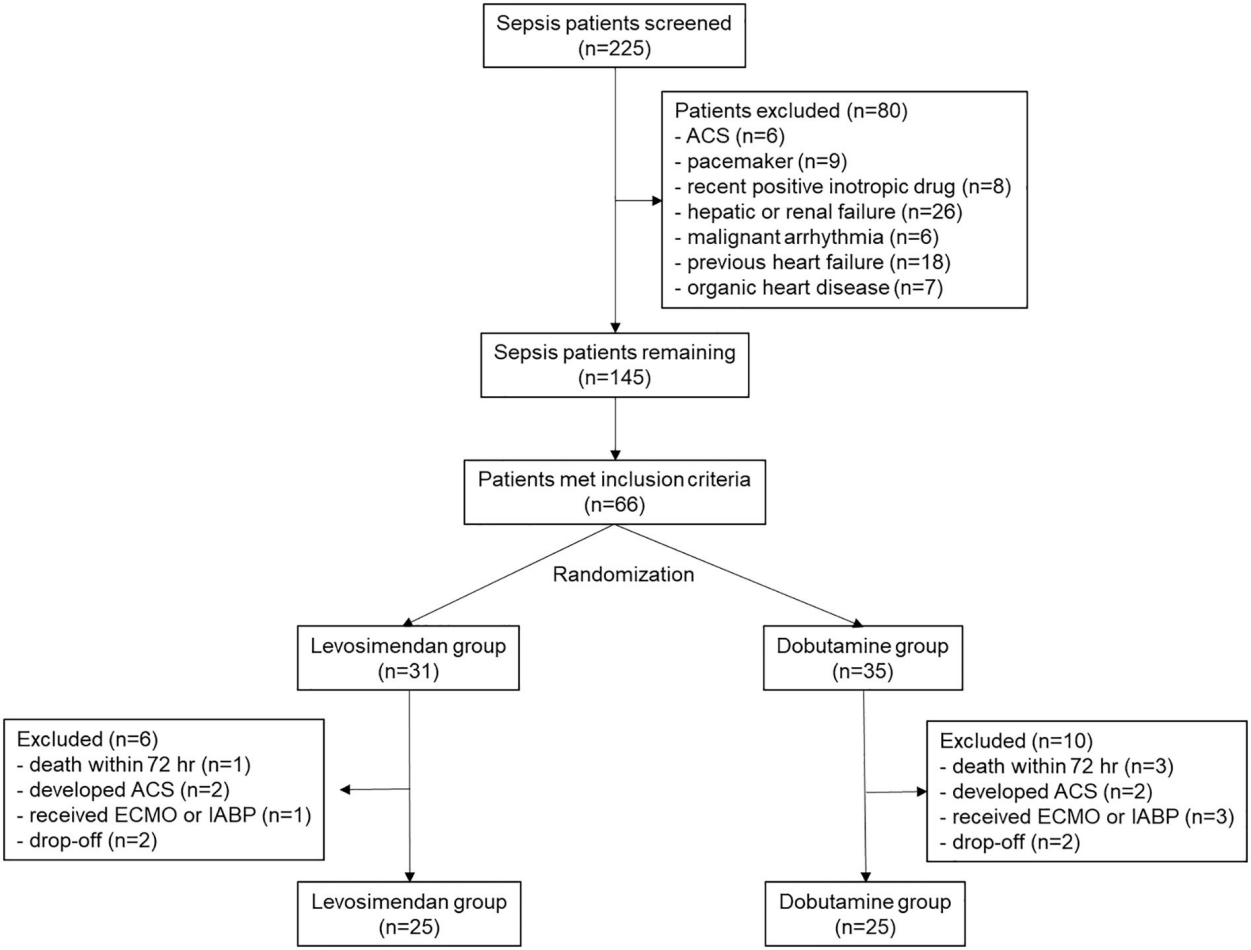
CONSORT flow diagram.

A total of 225 sepsis or septic shock patients were screened. Finally, 50 patients were analyzed, with 25 in both the levosimendan and the dobutamine groups. The CONSORT flowchart is shown in Figure 1. Their mean age was 76.4 (±12.3), with 28 (56%) male patients.

The baseline clinical characteristic comparisons showed no statistically significant differences between the two groups (Table 1). The baseline cardiac function measurements, blood pressure, laboratory test results, and PiCCO examinations were comparable between the two groups (Table 2).

**Table 1:** Baseline clinical characteristic comparisons between two groups.

Characteristics	Levosimendan group (*n* = 25)	Dobutamine group (*n* = 25)	t/*χ*^2^	*P*
Age, years, M ± SD	76.8 ± 14.0	76.1 ± 10.7	0.182	0.857
Sex, *n*			0.325	0.569
Male	15	13		
Female	10	12		
Height, cm, M ± SD	165.4 ± 8.6	164.1 ± 9.3	0.506	0.615
Weight, kg, M ± SD	55.4 ± 9.4	56.0 ± 9.8	−0.192	0.849
APACHE II score, M ± SD	22.9 ± 4.3	23.3 ± 5.0	0.305	0.761
SOFA score, M ± SD	11.5 ± 3.0	11.8 ± 2.3	0.422	0.675
Site of primary infections, *n*			0.121	0.941
Lung	17	16		
Abdomen	5	6		
Urinary tract	3	3		

M ± SD, mean ± standard deviation.

APACHE II, acute physiology, and chronic health evaluation II; SOFA, sequential organ failure assessment.

**Table 2:** Baseline mean arterial pressure, lactate, doses of norepinephrine, cardiac function measurements, laboratory test results, and pulse index continuous cardiac output examination comparisons between two groups.

Characteristics	Levosimendan group (*n* = 25)	Dobutamine group (*n* = 25)	t/Z	*P*
MAP, mmHg	65.3 ± 5.4	67.9 ± 7.2	−1.465	0.149
Lactate, mmol/L	6.6 ± 1.9	7.1 ± 1.5	−0.955	0.344
NED, μg/kg/min	0.5 ± 0.2	0.6 ± 0.2	−0.719	0.475
Cardiac function measurements				
LVEF, %	36.8 ± 4.0	36.7 ± 5.5	0.088	0.930
cTnI, ng/ml	1.4 ± 1.1	1.5 ± 0.9	−0.254	0.801
BNP, pg/ml	1,198.8 ± 486.3	1,283.0 ± 474.5	−0.620	0.538
Laboratory results				
WBC, ×10^9^ /L	13.8 ± 4.7	14.6 ± 4.9	−0.550	0.585
CRP, mg/L	106.1 (62.4, 160.0)	123.0 (72.0, 156.0)	−.0650	0.516
PCT, ng/ml	2.3 (1.0, 12.2)	7.8 (2.5, 12.2)	−1.330	0.184
PiCCO measurements				
CI, L/min/m^2^	2.5 ± 0.6	2.5 ± 0.5	0.173	0.863
GEDVI, ml/m^2^	699.7 ± 83.9	717.8 ± 85.9	−0.756	0.453
EVLWI, ml/kg	9.8 ± 1.4	10.1 ± 1.4	−0.721	0.474
SVRI, Dyn*s*cm^−5^m^2^	1,585.5 ± 299.1	1,631.0 ± 270.1	−0.564	0.575
HR, bpm	103.2 ± 13.7	105.6 ± 17.6	−0.547	0.587
CVP, mmHg	6.7 ± 2.4	7.1 ± 2.6	−0.562	0.577

All data are presented as mean ± standard deviation, except CRP and PCT, which are presented as median (interquartile range).

BNP, B-type natriuretic peptide; CI, cardiac index; CRP, C-reactive protein; cTnI, cardiac troponin I; CVP, central venous pressure; Extravascular lung water index (EVLWI) GEDVI, global end-diastolic volume; HR, heart rate; LVEF, left ventricular ejection fracture; MAP, mean arterial pressure; NED, doses of norepinephrine; PCT, procalcitonin; PiCCO, pulse index continuous cardiac output; SVRI, systemic vascular resistance index; WBC, white blood cell.

### Comparisons of cardiac function measurements, MAP, doses of norepinephrine, and lactate level after treatments between two groups

**Figure 2:**
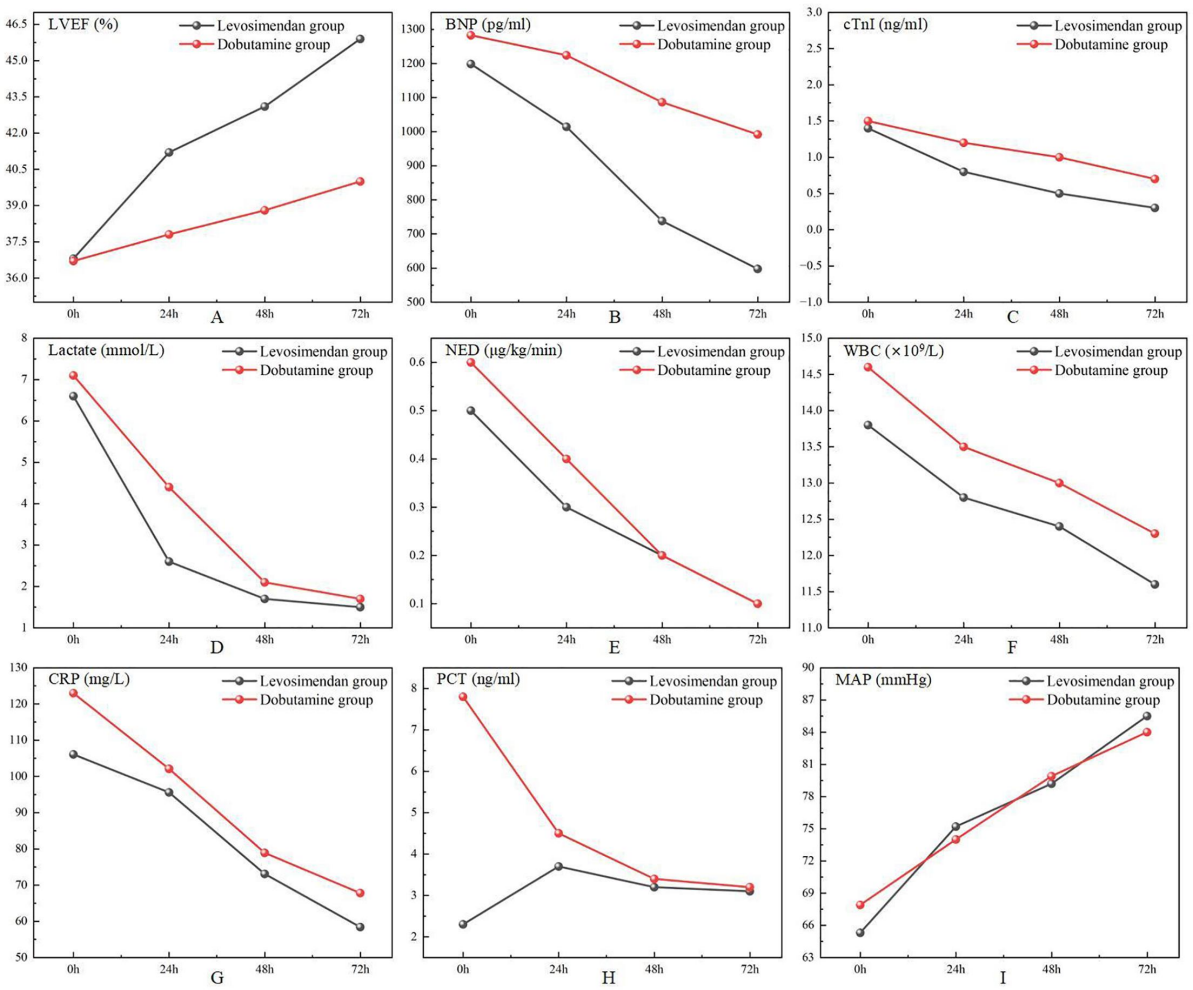
Comparison of cardiac function measurements, doses of norepinephrine, lactate, laboratory test results, and mean arterial pressure after treatments between two groups.

As shown in Figure 2; Table 3, following treatment initiation, the LVEF level in the levosimendan group was significantly higher than in the dobutamine group (*P* < 0.05). In contrast, levels of cTnI, BNP, lactate, and NED were significantly lower in the levosimendan group compared with the dobutamine group (all *P* < 0.05). However, the two groups had no significant difference in MAP (*P* > 0.05).

**Table 3:** Comparison of cardiac function measurements, mean arterial pressure, lactate, and doses of norepinephrine after treatments between two groups.

Measurements	Hours after treatment initiation			F	*P*
	24 h	48 h	72 h		
LVEF, %				6.480	0.014
Levosimendan group	41.2 ± 3.9	43.1 ± 4.0.	45.9 ± 4.2		
Dobutamine group	37.8 ± 5.6	38.8 ± 5.3	40.0 ± 5.5		
cTnI, ng/ml				4.218	0.045
Levosimendan group	0.8 ± 0.4	0.5 ± 0.2	0.3 ± 0.2		
Dobutamine group	1.2 ± 0.8	1.0 ± 0.7	0.7 ± 0.5		
BNP, pg/ml				7.105	0.010
Levosimendan group	1,014.3 ± 397.7	738.2 ± 331.7	597.5 ± 330.5		
Dobutamine group	1,224.3 ± 454.4	1,086.4 ± 437.4	991.9 ± 418.3		
MAP, mmHg				0.034	0.854
Levosimendan group	75.2 ± 3.4	79.2 ± 5.3	85.5 ± 7.4		
Dobutamine group	74.0 ± 5.8	79.9 ± 6.7	84.0 ± 6.1		
NED (μg/kg/min)				6.153	0.017
Levosimendan group	0.3 ± 0.1	0.2 ± 0.1	0.1 ± 0.1		
Dobutamine group	0.4 ± 0.1	0.2 ± 0.1	0.1 ± 0.1		

All data are presented as mean ± standard deviation.

BNP, B-type natriuretic peptide; cTnI, cardiac troponin I; CVP, central venous pressure; LVEF, left ventricular ejection fracture; MAP, mean arterial pressure; NED, doses of norepinephrine.

### Comparisons of PiCCO measurements after treatments between two groups

**Figure 3:**
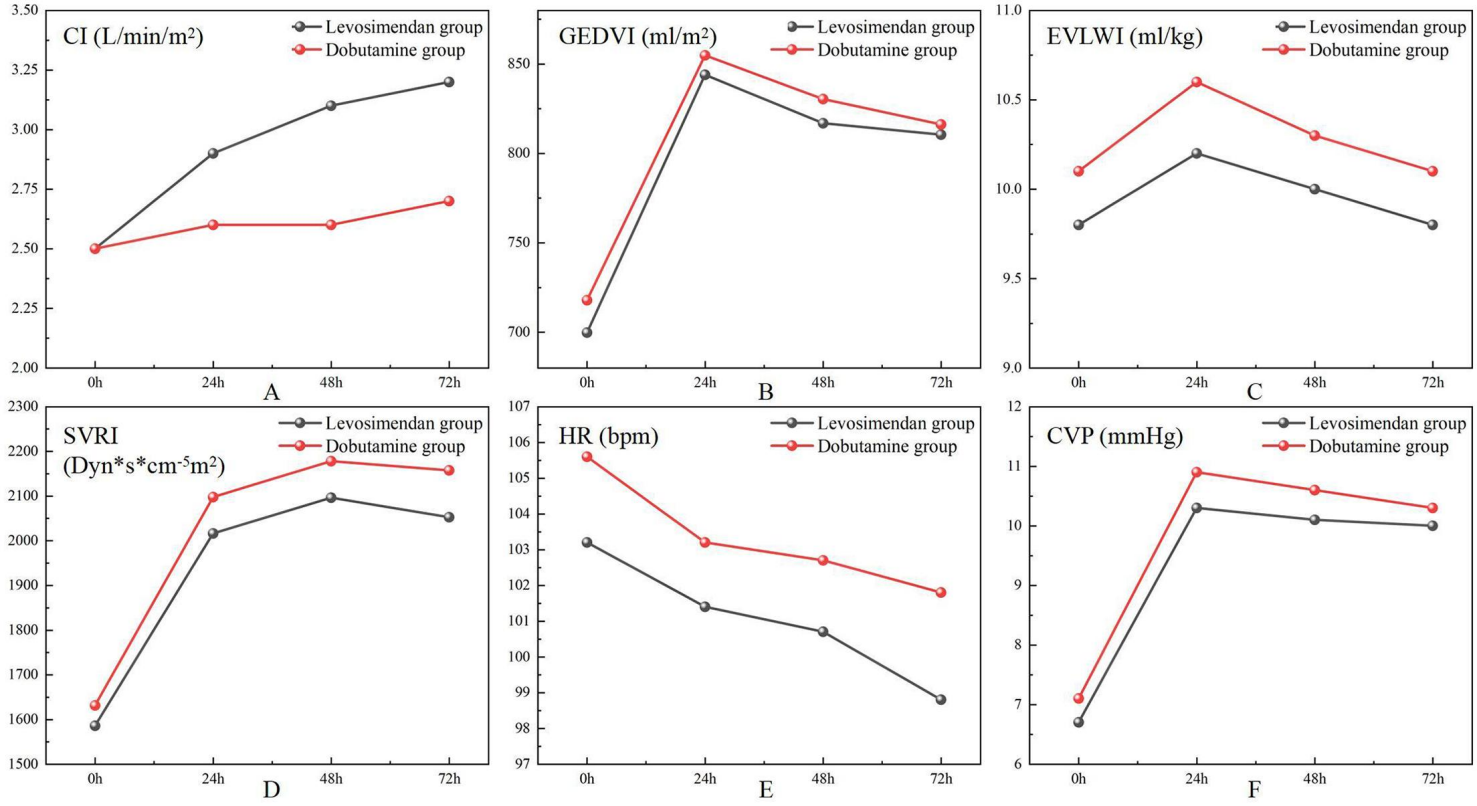
Comparison of measurements on the pulse index continuous cardiac output measurements after treatments between two groups.

As shown in Figure 3; Table 4, the CI was significantly higher in the levosimendan group compared to the dobutamine group after treatment (*P* < 0.05). No significant differences were observed between groups in GEDVI, EVLWI, SVRI, HR, or CVP (*P* > 0.05).

**Table 4:** Comparison of measurements on the pulse index continuous cardiac output measurements after treatments between two groups.

Measurements	Hours after treatment initiation			F	*P*
	24 h	48 h	72 h		
CI, L/min/m^2^				4.443	0.040
Levosimendan group	2.9 ± 0.5	3.1 ± 0.6	3.2 ± 0.6		
Dobutamine group	2.6 ± 0.5	2.6 ± 0.5	2.7 ± 0.5		
GEDVI, ml/m^2^				1.331	0.254
Levosimendan group	844.0 ± 63.1	816.9 ± 67.8	810.5 ± 44.5		
Dobutamine group	854.9 ± 64.9	830.4 ± 56.0	816.2 ± 62.7		
EVLWI, ml/kg				1.392	0.244
Levosimendan group	10.2 ± 0.9	10.0 ± 0.8	9.8 ± 1.0		
Dobutamine group	10.6 ± 1.3	10.3 ± 0.9	10.1 ± 1.3		
SVRI, Dyn*s*cm^−5^m^2^				1.868	0.178
Levosimendan group	2,016.2 ± 382.5	2,096.3 ± 296.3	2,052.5 ± 208.4		
Dobutamine group	2,097.6 ± 266.5	2,177.9 ± 268.4	2,157.3 ± 269.3		
HR, bpm				3.951	0.053
Levosimendan group	101.4 ± 7.4	100.7 ± 8.1	98.8 ± 5.9		
Dobutamine group	103.2 ± 5.1	102.7 ± 6.5	101.8 ± 6.2		
CVP, mmHg				1.280	0.264
Levosimendan group	10.3 ± 1.8	10.1 ± 2.2	10.0 ± 2.2		
Dobutamine group	10.9 ± 2.0	10.6 ± 1.8	10.3 ± 2.1		

All data are presented as mean ± standard deviation.

CI, cardiac index; CVP, central venous pressure; EVLWI, Extravascular lung water index; GEDVI, global end-diastolic volume; HR, heart rate; SVRI, systemic vascular resistance index.

### Comparison of laboratory test results after treatments between two groups

As shown in Figure 2; Table 5, post-treatment levels of WBC, CRP, and PCT did not differ significantly between the levosimendan and dobutamine groups (*P* > 0.05).

**Table 5:** Comparison of laboratory test results after treatments between two groups.

Time	Laboratory tests	Levosimendan group		t/Z	*P*
		Dobutamine group			
24 h	WBC, ×10^9^ /L	12.8 ± 3.3	13.5 ± 3.5	−0.749	0.457
	CRP, mg/L	95.6 (63.4, 133.0)	102.1 (76.4, 134.8)	−0.621	0.535
	PCT, ng/ml	3.7 (0.9, 9.4）	4.5 (2.4, 10.4)	−0.902	0.367
48 h	WBC, ×10^9^ /L	12.4 ± 2.2	13.0 ± 3.8	−0.634	0.529
	CRP, mg/L	73.1 (5.7, 110.0）	78.9 (47.3, 115.2)	−0.039	0.969
	PCT, ng/ml	3.2 (0.6, 6.1）	3.4 (2.1, 6.8)	−1.087	0.277
72 h	WBC, ×10^9^ /L	11.6 ± 4.6	12.3 ± 3.0	−0.608	0.547
	CRP, mg/L	58.4 (43.0, 100.4）	67.8 (39.3, 100.6)	−0.243	0.808
	PCT, ng/ml	3.1 (1.3, 5.6）	3.2 (1.3, 5.6)	−0.864	0.388

WBC are presented as mean ± standard deviation, and CRP and PCT are presented as median (interquartile range).

CRP, C-reactive protein; PCT, procalcitonin; WBC, white blood cell.

### Comparisons of clinical outcomes and adverse reactions between two groups

There were no significant differences in the length of ICU days, duration of mechanical ventilation, death at 28 days, SOFA scores after 72 h of treatments, and APACHE II scores after 72 h of treatments, as well as the incidence of adverse reactions, between the two groups (*P* > 0.05) (Table 6).

**Table 6:** Comparison of clinical outcomes and adverse reactions between two groups.

Measurements	Levosimendan group	Dobutamine group	t/*χ*^2^	*P*
Clinical outcomes				
APACHE II score at 72 h	18.5 ± 4.5	18.7 ± 3.6	−0.174	0.862
SOFA score at 72 h	9.2 ± 2.8	9.4 ± 2.0	0.233	0.817
Duration of mechanical ventilation, days	11.5 ± 4.8	12.2 ± 4.8	−0.473	0.638
Length of ICU stay, days	19.6 ± 5.9	18.5 ± 6.7	0.651	0.518
Survival at day 28, *n*	13	11	0.321	0.571
Adverse reactions, n			0.092	0.762
Hypotension	4	2		
Arrhythmia	1	2		

Data are presented as mean ± standard deviation unless specified.

## Discussion

Septic cardiomyopathy is a common complication of severe sepsis and septic shock and is a reversible myocardial dysfunction. Its pathogenesis is not fully understood and can result from myocardial damage from bacterial toxins and various cytokines (11). The optimal management strategy is unclear. In the present study, we showed that levosimendan treatment could provide better cardiac function, hemodynamic stability, and tissue perfusion than dobutamine treatment without increasing the incidences of adverse reactions. Levosimendan could be a treatment option in this patient population.

There are no universally accepted diagnostic criteria for septic cardiomyopathy; however, most researchers agree on the following defining features (10): (1) acute and reversible myocardial dysfunction, with gradual recovery of cardiac function within 7–10 days after disease onset; (2) left ventricular dilatation; (3) bilateral systolic and/or diastolic dysfunction; (4) poor responsiveness to fluid resuscitation and catecholamines; and (5) exclusion of acute myocardial ischemia resulting from coronary artery stenosis.

The primary therapeutic goal in septic cardiomyopathy is to improve cardiac function. In patients who have completed initial volume resuscitation but continue to exhibit MAP below 65 mmHg despite high-dose vasopressor therapy, positive inotropic agents are required to enhance myocardial contractility, increase cardiac output, and improve tissue perfusion. Dobutamine is currently the first-line inotropic agent recommended for patients with septic shock and concurrent cardiac dysfunction (12). However, several subsequent studies have failed to demonstrate that dobutamine improves microcirculatory function or long-term prognosis in patients with septic shock (13, 14).

The results of our study suggested that the addition of levosimendan to the standard treatment of patients with septic cardiomyopathy improved cardiac function and reduced myocardial injury better than dobutamine. Our results were similar to previous reports from Sun et al. (15) and Tsolaki et al. (16), as well as a meta-analysis of clinical trials of levosimendan vs. dobutamine in sepsis-associated cardiac insufficiency conducted by Liu et al. (9). The benefits of levosimendan might be related to the following mechanisms: (1) in sepsis/septic shock, β-adrenergic responsiveness is reduced, and catecholamines increase the risk of arrhythmias and myocardial oxygen consumption (17); (2) levosimendan increases the sensitivity of cardiomyocytes to calcium ions by altering the conformation of troponin C, which increases the calcium load and cyclic adenosine monophosphate levels in the absence of an increase in intracellular calcium loading and myocardial contractility and does not cause severe ventricular arrhythmias at therapeutic doses (18, 19); (3) levosimendan improves diastolic function of the heart by improving the diastolic flow velocity ratio, shortening the diastolic phase, and improving diastolic filling (20). In contrast, dobutamine improves only the systolic function, with insignificant improvement in diastolic function (21); (4) levosimendan has antioxidant activity, which can inhibit the release of oxygen free radicals through neutrophils and attenuate the damage of oxygen free radicals to mitochondria, thus reducing myocardial injury (22). Meanwhile, levosimendan can also inhibit the release of various cytokines and minimize their inhibitory effects on the myocardium (20).

The present study showed that, after treatments, hemodynamic measurements improved to varying degrees in both groups. In particular, levosimendan decreased lactate levels more rapidly within the first 24 h and reduced the norepinephrine dose faster than dobutamine. Similar results have been reported in previous studies. Hajjej et al. found that levosimendan could clear lactate better than dobutamine (23). Morelli et al. found that levosimendan improved sublingual microcirculation in patients with septic shock (24). Meng et al. found that levosimendan reduced extravascular lung water and lactate levels and improved tissue perfusion better than dobutamine (25).

In previous animal experiments (26), levosimendan was demonstrated to have anti-inflammatory activity and could prevent sepsis-induced multi-organ dysfunction. However, in the present study, we did not find any significant differences when comparing the laboratory inflammatory measurements of the two groups of patients before and after treatments. During sepsis, the inflammatory cytokines could be affected by the patient's underlying disease, the source and severity of the infection, and treatments, including antibiotics or steroids. In addition, dobutamine treatment might alter cytokine levels, contributing to the non-significant difference between the two groups.

There has been ongoing controversy regarding whether levosimendan reduces mortality and improves prognosis in patients with sepsis. Gordan et al. reported no difference in morbidity or mortality when levosimendan was administered to septic patients compared to placebo (8). In contrast, Zangrillo et al. observed a reduction in mortality in patients with severe sepsis or septic shock treated with levosimendan (27). A more recent meta-analysis concluded that levosimendan did not significantly affect mortality in this patient population (9).

In the present study, SOFA score at 72 h post-treatment, APACHE II score at 72 h post-treatment, duration of mechanical ventilation, length of ICU stay, and 28-day mortality had no statistically significant inter-group differences, suggesting that levosimendan did not reduce disease severity or improve short-term prognosis compared with dobutamine.

Additionally, there was no statistically significant difference in the incidence of adverse reactions between the two groups, indicating that levosimendan was safe for use in treating patients with septic cardiomyopathy.

This study has several limitations. It was a single-center investigation with a small sample size. Cardiac diastolic function was not assessed, and levosimendan and dobutamine were administered for a short duration. Therefore, large-scale, prospective, multicenter, randomized controlled trials are needed to further evaluate the clinical efficacy of levosimendan in patients with septic cardiomyopathy.

In conclusion, adding levosimendan to standard treatment in patients with septic cardiomyopathy resulted in greater cardiac function, myocardial protection, hemodynamic stability, and tissue perfusion compared with dobutamine, without an increase in significant drug-related adverse reactions. Further studies are warranted to confirm these findings and to investigate the potential long-term effects of levosimendan on clinical outcomes in this patient population.

## Data Availability

The original contributions presented in the study are included in the article/Supplementary Material, further inquiries can be directed to the corresponding author.

## References

[1] Singer M Deutschman CS Seymour CW Shankar-Hari M Annane D Bauer M The third international consensus definitions for sepsis and septic shock (sepsis-3). JAMA. (2016) 315(8):801–10. 10.1001/jama.2016.0287 26903338 PMC4968574

[2] Beesley SJ Weber G Sarge T Nikravan S Grissom CK Lanspa MJ Septic cardiomyopathy. Crit Care Med. (2018) 46(4):625–34. 10.1097/CCM.0000000000002851 29227368

[3] Ravikumar N Sayed MA Poonsuph CJ Sehgal R Shirke MM Harky A. Septic cardiomyopathy: from basics to management choices. Curr Probl Cardiol. (2021) 46(4):100767. 10.1016/j.cpcardiol.2020.100767 33388489

[4] Dubin A Mugno M. The effects of dobutamine in septic shock: an updated narrative review of clinical and experimental studies. Medicina (Kaunas). (2024) 60(5):751. 10.3390/medicina60050751 38792934 PMC11123338

[5] Annane D Ouanes-Besbes L de Backer D Du B Gordon AC Hernández G A global perspective on vasoactive agents in shock. Intensive Care Med. (2018) 44(6):833–46. 10.1007/s00134-018-5242-5 29868972

[6] Antoniades C Tousoulis D Koumallos N Marinou K Stefanadis C. Levosimendan: beyond its simple inotropic effect in heart failure. Pharmacol Ther. (2007) 114(2):184–97. 10.1016/j.pharmthera.2007.01.008 17363065

[7] Gordon AC Perkins GD Singer M McAuley DF Orme RM Santhakumaran S Levosimendan for the prevention of acute organ dysfunction in sepsis. N Engl J Med. (2016) 375(17):1638–48. 10.1056/NEJMoa1609409 27705084

[8] Antcliffe DB Santhakumaran S Orme RML Ward JK Al-Beidh F O'Dea K Levosimendan in septic shock in patients with biochemical evidence of cardiac dysfunction: a subgroup analysis of the LeoPARDS randomised trial. Intensive Care Med. (2019) 45(10):1392–400. 10.1007/s00134-019-05731-w 31428804

[9] Liu DH Ning YL Lei YY Chen J Liu YY Lin XF Levosimendan versus dobutamine for sepsis-induced cardiac dysfunction: a systematic review and meta-analysis. Sci Rep. (2021) 11(1):20333. 10.1038/s41598-021-99716-9 34645892 PMC8514594

[10] L'Heureux M Sternberg M Brath L Turlington J Kashiouris MG. Sepsis-induced cardiomyopathy: a comprehensive review. Curr Cardiol Rep. (2020) 22(5):35. 10.1007/s11886-020-01277-2 32377972 PMC7222131

[11] Hollenberg SM Singer M. Pathophysiology of sepsis-induced cardiomyopathy. Nat Rev Cardiol. (2021) 18(6):424–34. 10.1038/s41569-020-00492-2 33473203

[12] Evans L Rhodes A Alhazzani W Antonelli M Coopersmith CM French C Surviving sepsis campaign: international guidelines for management of sepsis and septic shock 2021. Intensive Care Med. (2021) 47(11):1181–247. 10.1007/s00134-021-06506-y 34599691 PMC8486643

[13] Hernandez G Bruhn A Luengo C Regueira T Kattan E Fuentealba A Effects of dobutamine on systemic, regional and microcirculatory perfusion parameters in septic shock: a randomized, placebo-controlled, double-blind, crossover study. Intensive Care Med. (2013) 39(8):1435–43. 10.1007/s00134-013-2982-0 23740284

[14] Gao F Zhang Y. Inotrope use and intensive care unit mortality in patients with cardiogenic shock: an analysis of a large electronic intensive care unit database. Front Cardiovasc Med. (2021) 8:696138. 10.3389/fcvm.2021.696138 34621796 PMC8490645

[15] Sun T Zhang N Cui N Wang SH Ding XX Li N Efficacy of levosimendan in the treatment of patients with severe septic cardiomyopathy. J Cardiothorac Vasc Anesth. (2023) 37(3):344–9. 10.1053/j.jvca.2022.10.032 36473763

[16] Tsolaki V Zakynthinos GE Papanikolaou J Vazgiourakis V Parisi K Fotakopoulos G Levosimendan in the treatment of patients with severe septic cardiomyopathy. Life (Basel). (2023) 13(6):1346. 10.3390/life13061346 37374128 PMC10303716

[17] Shahreyar M Fahhoum R Akinseye O Bhandari S Dang G Khouzam RN. Severe sepsis and cardiac arrhythmias. Ann Transl Med. (2018) 6(1):6. 10.21037/atm.2017.12.26 29404352 PMC5787725

[18] Chang W Xie JF Xu JY Yang Y. Effect of levosimendan on mortality in severe sepsis and septic shock: a meta-analysis of randomised trials. BMJ Open. (2018) 8(3):e019338. 10.1136/bmjopen-2017-019338 29602841 PMC5884355

[19] Kolseth SM Rolim NP Salvesen Ø Nordhaug DO Wahba A Høydal MA. Levosimendan improves contractility *in vivo* and *in vitro* in a rodent model of post-myocardial infarction heart failure. Acta Physiol (Oxf). (2014) 210(4):865–74. 10.1111/apha.12248 24495280

[20] Yang F Zhao LN Sun Y Chen Z. Levosimendan as a new force in the treatment of sepsis-induced cardiomyopathy: mechanism and clinical application. J Int Med Res. (2019) 47(5):1817–28. 10.1177/0300060519837103 30958071 PMC6567749

[21] Barraud D Faivre V Damy T Welschbillig S Gayat E Heymes C Levosimendan restores both systolic and diastolic cardiac performance in lipopolysaccharide-treated rabbits: comparison with dobutamine and milrinone. Crit Care Med. (2007) 35(5):1376–82. 10.1097/01.CCM.0000261889.18102.84 17414729

[22] Hasslacher J Bijuklic K Bertocchi C Kountchev J Bellmann R Dunzendorfer S Levosimendan inhibits release of reactive oxygen species in polymorphonuclear leukocytes *in vitro* and in patients with acute heart failure and septic shock: a prospective observational study. Crit Care. (2011) 15(4):R166. 10.1186/cc10307 21749676 PMC3387603

[23] Hajjej Z Meddeb B Sellami W Labbene I Morelli A Ferjani M. Effects of levosimendan on cellular metabolic alterations in patients with septic shock: a randomized controlled pilot study. Shock. (2017) 48(3):307–12. 10.1097/SHK.0000000000000851 28234790 PMC5516668

[24] Morelli A Donati A Ertmer C Rehberg S Lange M Orecchioni A Levosimendan for resuscitating the microcirculation in patients with septic shock: a randomized controlled study. Crit Care. (2010) 14(6):R232. 10.1186/cc9387 21182783 PMC3219978

[25] Meng JB Hu MH Lai ZZ Ji CL Xu XJ Zhang G Levosimendan versus dobutamine in myocardial injury patients with septic shock: a randomized controlled trial. Med Sci Monit. (2016) 22:1486–96. 10.12659/MSM.898457 27138236 PMC4861009

[26] Tsao CM Li KY Chen SJ Ka SM Liaw WJ Huang HC Levosimendan attenuates multiple organ injury and improves survival in peritonitis-induced septic shock: studies in a rat model. Crit Care. (2014) 18(6):652. 10.1186/s13054-014-0652-4 25432865 PMC4274679

[27] Zangrillo A Putzu A Monaco F Oriani A Frau G De Luca M Levosimendan reduces mortality in patients with severe sepsis and septic shock: a meta-analysis of randomized trials. J Crit Care. (2015) 30(5):908–13. 10.1016/j.jcrc.2015.05.017 26093802

